# A Population-Based Surveillance Study on the Epidemiology of Hepatitis C in Estonia

**DOI:** 10.3390/medicina54010009

**Published:** 2018-03-25

**Authors:** Kairi Mansberg, Karin Kull, Riina Salupere, Tiina Prükk, Benno Margus, Toomas Kariis, Triin Remmel, Külliki Suurmaa, Kristi Ott, Krista Jaago, Jelena Šmidt

**Affiliations:** 1 Department of Gastroenterology, Tartu University Hospital, University of Tartu, 51014 Tartu, Estonia; karin.kull@kliinikum.ee (K.K.); riina.salupere@kliinikum.ee (R.S.); tiina.prykk@kliinikum.ee (T.P.); 2 Internal Disease Clinic, Tartu University Hospital, University of Tartu, 51014 Tartu, Estonia; 3 Department of Gastroenterology, East Tallinn Central Hospital, 10138 Tallinn, Estonia; benno.margus@itk.ee (B.M.); toomas.kariis@itk.ee (T.K.); triin.remmel@itk.ee (T.R.); 4 Department of Gastroenterology and Infectios Diseases Clinic, West Tallinn Central Hospital, 10138 Tallinn, Estonia; ksuurmaa@gmail.com (K.S.); kristi.ott@ltkh.ee (K.O.); 5 Internal Disease Clinic, Pärnu Hospital, 80010 Pärnu, Estonia; krista.jaago@ph.ee; 6 Internal Disease Clinic, East Viru Central Hospital, 31024 Kohtla-Järve, Estonia; jelena.smidt@ivkh.ee

**Keywords:** hepatitis C virus, epidemiology, hepatitis C risk factors, genotypes

## Abstract

*Background and objective:* The hepatitis C virus (HCV)-infected patients serve as a reservoir for transmission of the disease to others and are at risk of developing chronic hepatitis C, cirrhosis, and hepatocellular carcinoma. Although the epidemiological data of high rate HCV infection have been obtained in many countries, such data are insufficient in Estonia. Therefore, the aim of the study was to analyze country-specific data on HCV patients. *Materials and methods:* Data about age, gender, diagnosis, possible risk factors, coinfections, HCV genotypes, liver fibrosis stages and extrahepatic manifestations were collected from 518 patients. *Results:* The most common risk factors for hepatitis C were injection drug use and tattooing in the 30–39 and 40–49 year age groups, and blood transfusion in the 50–59 and 60–69 year age groups. The other risk factors established were profession-related factors and sexual contact. The prevailing viral genotype among the HCV infected patients was genotype 1 (69% of the patients) followed by genotype 3 (25%). Genotypes 1 and 3 correlated with blood transfusions before 1994, drug injections and tattooing. *Conclusions:* Our study provides the best representation of genotype distribution across Estonia. As a result of the study, valuable data has been collected on hepatitis C patients in Estonia.

## 1. Introduction

The hepatitis C virus (HCV) is responsible for a large proportion of chronic liver disease worldwide. The global prevalence of chronic hepatitis C is estimated at an average of 3% [1,2]. Chronic hepatitis may progress to fibrosis, cirrhosis and hepatocellular carcinoma (HCC). It has been estimated that HCV accounts for 27% of cirrhosis and 25% of HCC worldwide [3,4]. The rate of hepatitis progression is influenced by several risk factors, such as age at infection, gender, coinfection with human immunodeficiency virus (HIV) and hepatitis B virus (HBV), and alcohol consumption. The prevalence of cirrhosis has doubled and the prevalence of HCC has increased 20-fold during the last decade [5,6]. According to the World Health Organization (WHO), two-thirds of liver transplants are due to HCV infection and a US consensus conference named HCV infection the primary reason for liver transplantation [7].

In Europe, the lowest anti-HCV prevalence occurs in Northern Europe (<0.5%), and the highest prevalence occurs in Romania, Italy, Greece, and Russia (>3%) [8]. In the Baltic countries, relevant data have been available from Lithuania, with an anti-HCV prevalence of 2.8% [9]. The epidemiological data on HCV-infection are scant in Estonia. The prevalence of anti-HCV for 2013 as estimated by expert consensus was between 1.5% and 2.4% [10].

Multiple genotype studies confirm that the most common genotype in Estonia is 1b, accounting for 75% of cases [11]. At the same time, there are no data about the routes of HCV transmission or about HCV patient data, nor is there relevant data about the prevalence of HCC or mortality related to HCV.

The aim of this investigator-initiated, population-based surveillance study was to analyze the following data on HCV patients in Estonia: Time of infection, possible routes of transmission and risk factors of HCV, coinfections (HIV, HBV), distribution of genotypes, alcohol consumption, related diseases, and antiviral treatment.

## 2. Experimental Section

All consecutive adult patients (19–81 years old) with acute hepatitis C, chronic hepatitis C, HCV-related cirrhosis and HCV-related HCC who visited a gastroenterologist or an infectionist between 1 February 2009 and 31 January 2010 were recruited into the hepatitis C virus investigator-initiated study (HCVIIS). The study included both outpatients and inpatients from all hospitals in Estonia where HCV-infected patients were treated (Tartu University Hospital, Pärnu Hospital, East Tallinn Central Hospital, West Tallinn Central Hospital and East Viru Central Hospital). Almost all gastroenterologists and infectionists (31 gastroenterologists and infectionists) who are in charge of HCV patients in Estonia participated in this population-based surveillance study.

Data were collected via a web-based electronic case report form (eCRF). The following data were registered: Date of birth, gender, diagnosis (acute hepatitis C, chronic hepatitis C, HCV-related cirrhosis, HCV-related hepatocellular carcinoma), time of diagnosis (newly diagnosed, previously diagnosed), possible risk factors and route of infection (injection drug use, profession related risk, blood transfusion before 1994 or after 1994, major surgical operations, tattooing, piercing, acupuncture, haemodialysis, known HCV-positive sexual contact), co-infection with HIV and/or hepatitis B, extrahepatic manifestations, alcohol consumption, related illnesses, HCV genotype, liver biopsy finding, and antiviral treatment.

The study was initiated and monitored by the Estonian Society of Gastroenterology. The study protocol and the informed consent form were submitted to the Ethics Committee of the University of Tartu, who approved the study (protocol No. 177/T-12). The work was carried out in accordance with the Code of Ethics of the World Medical Association (Declaration of Helsinki). All included patients provided written, informed consent. The study monitor had direct access to the investigators’ source documentation, in order to verify the consistency of the data recorded in the eCRF. The monitor was responsible for routine review of the eCRF entries at regular intervals, 4 times per year.

### Statistical Analysis

For statistical analyses, Stata Statistical Software, Release version 10.0 (StataCorp LP, College Station, TX, USA) software was used. Demographic characteristics were summarized using standard descriptive methods. Comparisons between the groups were made using the chi-square test and Fisher’s exact test. To analyze an age-based trend, the chi-square test was used. A two-tailed *p*-value less than 0.05 was considered statistically significant. The results are presented as percentages with the 95% confidence interval. 

## 3. Results

### 3.1. HCV Patients

Altogether, 518 Estonian patients (271 male, i.e., 52.7%, and 247 female, i.e., 47.7%) participated in the study. To study the risk factors and viral genotypes of the HCV patients in different age groups, the data of the patients were divided according to six age groups: Under 29 years, 30–39 years, 40–49 years, 50–59 years, 60–69 years, and over 70 years of age (Table 1).

The mean age of the HCV patients was 45.3 years (95% CI 43.5–47.1 years) for male patients and 48.2 years (95% CI 46.2–50.2 years) for female patients. HCV infection was diagnosed more frequently in the 40–49 year age group (26.9%) for men, and in the 50–59 year age group for women (22.2%). Age distribution between genders was statistically significant (*p* = 0.001) (Table 1).

A total of 196 patients out of the 518 had newly-diagnosed HCV liver disease during the study period. Among the 446 chronic hepatitis C patients, the disease was newly diagnosed in 169 patients (38%) and among the 65 HCV-related cirrhosis patients, the disease was newly diagnosed in 21 (32%).

The HCV patients (518) were referred to a gastroenterologist or infectionist by their family physician in 276 (53.3%) cases, or by other specialists/doctors in 145 (28%) cases. In 78 (15.1%) patients, anti-HCV positivity was established during prophylactic investigations studies (anonymous blood testing, occasional liver tests done for various reasons).

Four hundred and forty-six (86%) patients had chronic hepatitis C, 65 (13%) patients had HCV-related cirrhosis, and three (0.6%) had hepatitis C-related hepatocellular carcinoma. Acute hepatitis C was diagnosed in 4 (0.8%) patients out of the 518 during the study period. 

HCV-related extrahepatic manifestations (cryoglobulinemia and vasculitis, cutaneous porphyria, lichen planus) were diagnosed in 10 patients (1.9%). Cryoglobulinemia and vasculitis were diagnosed in six patients (1.1%). Cutaneous porphyria was diagnosed in one patient and three patients had lichen planus.

All patients were tested for HIV and hepatitis B virus (HBV). Coinfection with HIV and/or HBV was diagnosed in 22 patients (4.8%): HIV in 15, HBV in 7 and both HIV and HBV in 2 patients. HIV-HCV coinfection was most common in the younger age groups (up to 39 years).

According to our data, 68% of the patients were consuming alcohol. Alcohol drinkers differed in age (*p* = 0.021): Alcohol consumption was more common in the younger age group (up to 39 years) and in middle-aged patients (40–59 years). Heavier drinkers (>40 g/day) were in the 40–49 or below 29 year age groups. The 40–49 year age group also included most of the patients who used less than 40 g of alcohol per day.

Among the study subjects, the highest number, 361 (68%), had secondary or secondary special education, 114 (22%) had higher education, and 43 (8.3%) had primary education.

The relationship between education level and awareness of disease (HCV awareness) was not considered in this study.

### 3.2. Risk Factors

For 40% of HCV patients (222), the HCV transmission route was unknown. Two hundred and ninety-six of the 518 patients reported a possible risk factor while 37 patients reported two to three possible risk factors (Table 2). The possibility that HCV was acquired from blood transfusion before 1994 was reported most often (154, i.e., 28%).

People Who Injected Drugs (PWID) formed another predominant group (68 patients, 12.3%). The other risk factors reported in this study were profession-related factors (17 patients, 3%), sexual contact with a HCV-positive person (20 patients, 4%), and tattooing (42 patients, 8%). In the under-29 year age group, the most prevalent risk factors were PWID (34–50%), piercing (4–40%) and known sexual contact with a HCV-positive person (33–40%). Tattooing was the most common risk factor in the 30–39 and 40–49 year age groups. In the 40–49 year age group, the proportion of unknown risk factors was the highest (71 patients, 32%). Blood transfusion before 1994 was the main risk factor in the 50–59 and 60–69 year age groups (Table 2).

### 3.3. HCV Genotypes

**Figure 1:**
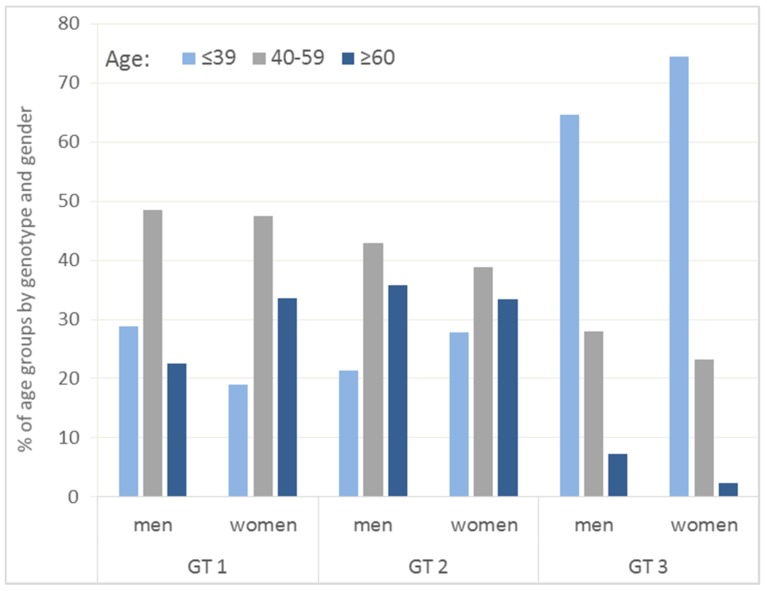
Percentage distribution of patients with HCV infection by HCV genotypes and age groups. Hepatitis C virus (HCV), genotypes (GT).

The HCV genotype was analyzed in 513 patients. The prevailing genotype was 1 (69% of the patients), followed by genotype 3 (25%) and genotype 2 (6%). Among the patients over 40 years of age (both 40–59 and 60+ year age groups), genotype 1 was the most prevalent (*p* < 0.001). Among the male patients under 40 years of age, genotype 3 was the most common (*p* < 0.001, Figure 1). Patients under 40 years of age experienced less genotype 1 than older patients (*p* < 0.001). One patient had a mixed viral genotype (genotype 1 and genotype 2).

Associations between risk factors and the HCV genotypes were studied. The following statistically-significant correlations were revealed (*p* < 0.05): for genotype 1, correlations with blood transfusion before 1994, PWID and tattooing; for genotype 3, the above correlations plus a correlation with sexual contact with a HCV-positive person. No statistically-significant correlations with risk factors were revealed for genotype 2.

### 3.4. Liver Fibrosis

Liver fibrosis data were available for 405 of the 446 chronic hepatitis patients. Liver percutaneous biopsy was a routine investigation in Estonia in the study period, and the METAVIR classification was used for histological evaluation. The majority of HCV patients with chronic hepatitis were classified as F4 (43, 10.6%), F3 (60, 14.8%), and F2 (94, 23.2%). Most of the hepatitis patients with early stage of liver disease (123, 34.4%) were classified as F1/F0.

### 3.5. Antiviral Treatment

In 2009, approximately 400 patients were treated for HCV infection (data from the Estonian Health Insurance Fund), and 245 patients of those who started treatment in 2009 were included in the present clinical study. At that time, treatment with peg-interferon alfa2a or 2b and ribavirin was available. During the study period, 245 (47.3%) patients started antiviral therapy; no treatment was initiated or had been previously provided to 273 (52.7%) patients.

## 4. Discussion

A large proportion (28%) of patients reported blood transfusion before 1994 as the possible route of infection. Blood transfusion in the past (before 1994) was the predominant risk factor for HCV infection in older age (50–69 years) and PWID was the predominant risk factor in the younger age cohorts. HCV has long been recognized as an essentially parenterally-transmitted infection in Estonia. Injection drug use as the main source of new HCV infection generally has a higher prevalence in younger age groups in Estonia, compared to other countries [12]. 

HCV/HIV coinfection is also common among active and former PWIDs, who may acquire both viruses from injecting drugs. In developed European countries and in the USA, PWIDs forms the most prevalent risk group, followed by the blood transfusion group. In Europe, the majority of PWIDs are infected with HCV, and globally 67% of the PWIDs are HCV-positive. One hypothesis is that countries where injection drug use is the main source of new HCV infection generally display higher prevalence among men of younger age groups, whereas countries historically related to an unsafe blood supply generally display higher prevalence among older age groups [13]. The data from the present epidemiological study may have introduced a selection bias in analysis as testing and treatment were likely not representative of hard-to-sample populations such as PWIDs. Sixteen patients out of the 22 with diagnosed HCV-HIV coinfection were involved in drug injection (PWID). At the same time, the data of 3750 subjects from the Estonian HIV Cohort Study database (E-HIV) were analyzed (42% of all reported HIV cases in Estonia), and HIV/HCV co-infection was diagnosed in 42% of the HIV cases. The prevalence of HIV–HCV coinfection was similar to that in Western European countries. According to the data of that study, 54% PWIDs vs. 30% sexually-infected patients were HCV-positive [14].

In Estonia, as well as in other countries, the majority of patients are not aware of when and how they have acquired HCV infection [15]. It could assumed that the risk behavior is displayed by people with a lower level of education. However, this issue should be clarified by research where both the level of education and the route of infection are co-analyzed. The fact that most of the study patients did not know how they had acquired HCV infection makes it impossible to confirm the above assumption. 

According to the literature, drinking more than 50 g/day of alcohol increases the risk of liver fibrosis in HCV patients 1.3-fold, compared to HCV-infected non-drinkers [16]. Among the study patients, 68.2% consumed alcohol. Alcohol consumption and HCV infection have a synergistic hepatotoxic effect and the coexistence of these factors increases the risk of advanced liver disease. Traditionally, the prevalence of HCV infection has been considered to be higher (up to 50%) in alcohol-consuming patients than in the general population [17]. In our study, 11 patients (2.12%) used more than 40 g of alcohol per day.

Several studies have analyzed the potential mechanisms of liver damage such as iron accumulation, altered cell-mediated immunity, increased oxidative stress, quasi-species generation and liver steatosis, through the combined effects of HCV and alcohol [18,19].

HCV has many genotypes and subtypes that have been isolated with different distribution patterns around the world. In Estonia, HCV genotypes were determined from the sera of HCV-positive patients in two periods: 1997–1998 and 2000–2004 [20]. The most prevalent HCV genotype in Estonia is genotype 1 (70%), followed by genotype 3 (24%) and genotype 2 (6%). The results of this study demonstrate that the distribution pattern of HCV genotypes has not changed since 1997.

As the hard-to-sample PWID population was under-represented in our study, we were not able to confirm the hypothesis that genotype distribution is rapidly changing, because of differences in the historical risk factors and the risk factors currently driving the HIV epidemic in Estonia.

Genotype distribution in 65 patients with HCV-related cirrhosis was the following: 43 patients had genotype 1, two patients had genotype 2 and 18 patients had genotype 3. The majority of cirrhosis patients belonged to the 40–59 year age group.

All three patients with the diagnosis of HCC had genotype 1, and two of them were diagnosed with HCC for the first time. Reports on the association between HCV genotype and HCC risk are inconsistent. However, a meta-analysis of 21 studies that calculated age-adjusted risk estimates reported that patients infected with HCV genotype 1b had an almost two-fold higher risk of developing HCC than patients with other HCV genotypes (a pooled relative risk of 1.78) [21,22].

Considering that in previous studies on HCV genotypes, genotype 1a was very scarce in Estonia (0.9–2%), we did not describe chronic hepatitis C subtypes in the HCVIIS study.

The finding of subtype 1b in patients older than 40 years with a history of medical interventions was not unexpected, and supports the opinion that 1b has occurred in Estonia since long ago. HCV subtypes 1b and 3a are predominant in Estonia, similar to in other Eastern European countries [23]. According to the Polaris data from a survey in 2015, the proportions of genotypes of the same magnitude are 71.7% for genotype 1 and 24.2% for genotype 3a. [24]

Immigration from endemic areas also diversifies the genotype distribution of HCV [25]; given the migration statistics of Estonia, we do not expect any changes in this regard.

In the database of the HCVIIS study, the HCV-related extrahepatic manifestations were reported in 10 (1.9%) patients out of 518. In our study, the most common extrahepatic manifestations were cryoglobulinemia and vasculitis, which were diagnosed in six patients (1.1%). Only a small fraction of patients with MC associated with HCV (<15%) have symptomatic disease. In our study, cryoglobulins were only detected in patients with vasculitis, which is why the percentage of these patients was relatively low. According to different studies, eighty percent of patients with cryoglobulinemia have HCV infection. HCV infection has also been associated with dermatological disorders, such as porphyria cutanea tarda and lichen planus [26,27,28].

In regard to the distribution of diagnoses, chronic hepatitis C is the most common, as expected. In the HCVIIS cohort of our study, very few patients had HCV-related hepatocellular carcinoma. Liver cirrhosis develops in 10–20% of patients with chronic hepatitis C over a disease period of approximately 20 years [29,30]. The rate of HCC among HCV-infected persons ranges from 1% to 3% over 30 years. HCV infection is associated with a 15- to 20-fold increase in the risk of HCC, compared with HCV-negative subjects in cross-sectional and case-control studies. In the United States, Latin America, Japan, and several regions of Europe, HCV is the leading cause of HCC [31]. HCV infects the immune system and endothelial cells, leading to immune dysfunction and chronic dysregulated angiogenesis. Both conditions are known risk factors for cancer [32].

The current study confirms that HCV infection is often diagnosed too late, in the HCV-related cirrhosis stage. The severity of liver disease in newly-diagnosed patients was remarkable: for 32% of HCV-related cirrhosis patients, it was the first liver disease diagnosis in their life.

## 5. Conclusions

Out of the 518 patients who participated in the present study, HCV infection was diagnosed more frequently in the 40–49 year age group for men and in the 50–59 year age group for women. For the majority of the patients (40%), the HCV transmission route was unknown. Tattooing was the most common risk factor in the 30–39 and 40–49 year age groups. Blood transfusion before 1994 was the main risk factor in the 50–59 and 60–69 year age groups. The other risk factors reported included profession-related risk, sexual contact and tattooing.

This study confirms the theory that the genotype distribution patterns vary with age, gender and coinfections, as well as across geographical areas and epidemiological groups. Based on previous studies, genotype 1 has remained unchanged in Estonia, followed by genotype 3.

According to the study, HCV liver disease is diagnosed too late in many patients—only in the HCV-related cirrhosis stage. Therefore, increasing the awareness of HCV among the population is crucial. Priorities need to be focused on preventing injection drug use and on improving access to HCV treatment, harm reduction counselling and testing, in order to identify HCV patients for medical evaluation and management.

## Figures and Tables

**Table 1:** Distribution of patients with HCV infection by age and gender.

Age (Years)							
Gender	≤29	30–39	40–49	50–59	60–69	≥70	Total
Men	46 (16.9)	60 (22.1)	73 (26.9)	40 (14.7)	29 (10.7)	23 (8.4)	271 (100.0)
Women	43 (17.4)	29 (11.7)	51 (20.6)	55 (22.2)	49 (19.8)	20 (8.1)	247 (100.0)
Total	89 (34.3)	89 (33.8)	124 (47.5)	95 (36.9)	78 (30.5)	43 (16.5)	518 (100.0)

Values are number (%); hepatitis C virus (HCV).

**Table 2:** Risk factors * of HCV according to age groups.

Risk Factors	Age (years)						Total	*p*
	≤29	30–39	40–49	50–59	60–69	≥70		
PWID	34 (50.0)	27 (39.7)	4 (5.9)	3 (4.4)	0 (0.0)	0 (0.0)	68 (100.0)	<0.001
Profession related risk	1 (5.9)	3 (17.6)	3 (17.6)	6 (35.3)	2 (11.8)	2 (11.8)	17 (100.0)	0.158
Blood transfusion before 1994	7 (4.5)	8 (5.2)	33 (21.4)	37 (24.0)	41 (26.6)	28 (18.2)	154 (100.0)	<0.001
Blood transfusion after 1994	0 (0.0)	5 (31.3)	3 (18.8)	1 (6.3)	6 (37.5)	1 (6.3)	16 (100.0)	0.213
Sexual contact with HCV patient	8 (40.0)	5 (25.0)	3 (15.0)	2 (10.0)	2 (10.0)	0 (0.0)	20 (100.0)	0.004
Tattooing	10 (23.8)	20 (47.6)	10 (23.8)	0 (0.0)	1 (2.4)	1 (2.4)	42 (100.0)	<0.001
Acupuncture	0 (0.0)	1 (100)	0 (0.0)	0 (0.0)	0 (0.0)	0 (0.0)	1 (100.0)	0.42
Piercing	4 (40.0)	2 (20.0)	2 (20.0)	1 (10.0)	1 (10.0)	0 (0.0)	10 (100.0)	0.056
Haemodialysis	0 (0.0)	0 (0.0)	0 (0.0)	0 (0.0)	0 (0.0)	1(100.0)	1 (100.0)	0.069
Unknown cause of infection	33 (14.9)	30 (13.5)	71 (32.0)	48 (21.6)	29 (13.1)	11 (5.0)	222 (100.0)	0.753

* Some patients reported several risk factors; Values are number (%); hepatitis C virus (HCV); people who injected drugs (PWID).
